# Artificial intelligence and ChatGPT literacy among surgical healthcare professionals: knowledge, attitudes, and perceived clinical utility

**DOI:** 10.3389/fdgth.2026.1766683

**Published:** 2026-05-19

**Authors:** Yasmin Safi, Saleh Abualhaj, Lina Alshadfan, Kinda Al-Kammash, Nadeen Al-Ramini, Leen Abuzaid, Saleh Abo Naji, Islam Alzayadneh, Mahmoud Al-Masri

**Affiliations:** 1 General Surgery Department, King Hussein Cancer Center, Amman, Jordan; 2 General Surgery Department, Faculty of Medicine, Al-Balqa Applied University, Al-Salt, Jordan; 3 Department of Pediatrics, Faculty of Medicine, Al Balqa Applied University, Salt, Jordan; 4 Department of General Surgery, Faculty of Medicine, Jordan University of Science and Technology, Irbid, Jordan; 5 Faculty of Medicine, Yarmouk University, Irbid, Jordan; 6 General Surgery Department, Istiklal Hospital Amman, Amman, Jordan; 7 Special Surgery Department, Faculty of Medicine, Mutah University, Karak, Jordan

**Keywords:** AI literacy, artificial intelligence, attitudes, ChatGPT, clinical utility, ethical awareness, surgical education

## Abstract

**Background:**

Artificial intelligence (AI) and large language models such as ChatGPT are increasingly applied in surgical education and practice. Successful integration requires healthcare professionals to have adequate literacy, perceived knowledge, and agreement in using these tools, alongside awareness of ethical considerations. Evidence on AI and ChatGPT literacy among surgical trainees and specialists remains limited.

**Objectives:**

This study aimed to evaluate AI and ChatGPT literacy, perceived knowledge, agreement in clinical application, attitudes, perceived barriers, and perceived clinical utility among surgical medical students, residents, and specialists.

**Methods:**

A cross-sectional survey was conducted among medical students (clinical years), surgical residents, and consultants/specialists in Jordan to assess AI literacy, ChatGPT literacy, attitudes, and barriers toward AI integration in surgical practice. Stratified random sampling with proportional allocation ensured representative participation across professional strata. A structured online questionnaire, including the Meta-AI Literacy Scale, ChatGPT literacy items, an AI exposure subscale, and attitude measures, was used. Subscale and overall scores were calculated, and internal consistency was high across all measures (Cronbach's *α* 0.846–0.930). Data were analyzed using R, with ANOVA or Kruskal–Wallis tests for group comparisons, chi-square tests for categorical associations, and significance set at *p* < 0.05.

**Results:**

Most participants lacked formal AI training (93.2%), but 80.9% had prior ChatGPT experience. AI exposure and perceived literacy scores were highest among students and residents, while consultants reported lower familiarity. Residents showed greater agreement in clinical AI application (MAIL_Use&Apply: 19.5 ± 5.2 vs. 17.4 ± 5.4; *p* = 0.014) and AI detection (MAIL_AI_Detect:9.0 ± 2.7 vs. 8.5 ± 2.2; *p* = 0.033) compared with consultants. Ethical awareness was high across all groups (MAIL_AI_Ethics:10.8 ± 2.9). AI was perceived as most useful for surgical planning, diagnostics, and robotic-assisted surgery, with trainees rating utility relatively higher than consultants (*p* < 0.01). Barriers included lack of training, cost, ethical concerns, and limited infrastructure.

**Conclusions:**

AI and ChatGPT literacy varies across professions, with younger trainees reporting relatively higher exposure, agreement, and perceived utility. Ethical awareness is generally high, and attitudes toward AI adoption are positive. Integrating structured AI education and addressing barriers are critical to safe, effective, and ethical adoption of AI in surgical education and practice.

## 1 Introduction

Artificial intelligence (AI) is rapidly transforming healthcare, offering novel tools for clinical decision-making, surgical planning, diagnostics, and medical education (1, 2). In surgery, AI applications such as computer vision, robotic-assisted surgery, and clinical decision support systems have the potential to enhance precision, improve patient outcomes, and optimize workflow efficiency (3, 4). Alongside these innovations, natural language processing tools like ChatGPT have emerged, providing clinicians and trainees with new opportunities for information synthesis, educational support, and documentation assistance (5). Despite these advances, effective integration of AI into clinical practice depends on healthcare professionals’ understanding, literacy, and confidence in using such tools.

AI literacy among healthcare providers is multifaceted, encompassing knowledge of fundamental concepts (e.g., machine learning, deep learning), the ability to detect AI-generated content, the capacity to apply AI appropriately in clinical scenarios, and awareness of ethical, legal, and regulatory considerations (6, 7). Studies suggest that while younger healthcare professionals and trainees may engage more frequently with AI tools, senior clinicians often demonstrate lower familiarity and confidence, potentially limiting adoption in practice (8, 9). Moreover, ethical concerns such as patient data privacy, algorithmic bias, and accountability remain critical barriers to safe and effective AI implementation (10, 11).

ChatGPT, a widely adopted large language model, exemplifies the growing role of AI in clinical and academic environments. Its applications range from assisting in literature review, drafting educational materials, and generating clinical summaries to supporting diagnostic reasoning (12, 13). However, proper utilization requires not only technical literacy but also critical evaluation skills to distinguish AI-generated content from human judgment and to understand limitations and ethical implications (14, 15).

In the context of surgical education and practice, assessing AI and ChatGPT literacy is crucial for identifying knowledge gaps, understanding attitudes toward adoption, and informing targeted training programs (16). Evidence on these topics, particularly in the Middle East and among surgical healthcare providers, remains limited. Understanding how professionals at different stages of training—students, residents, and consultants—perceive, utilize, and engage with AI can guide curriculum development, professional development initiatives, and policy decisions.

This study aimed to evaluate the perceived knowledge, exposure, and literacy of AI and ChatGPT among surgical healthcare professionals, to assess their ability to use and apply AI tools in clinical practice, and to explore attitudes, perceived barriers, and ethical awareness regarding AI integration. Additionally, it examined perceived clinical utility across surgical domains and investigated correlations among AI literacy, ChatGPT literacy, ethical awareness, and attitudes toward AI adoption. By doing so, the study seeks to inform strategies for integrating AI literacy into surgical education and practice, ensuring safe, effective, and ethically grounded adoption of emerging technologies.

## 2 Methods

### 2.1 Study design and setting

This study employed a cross-sectional survey design to assess AI literacy, ChatGPT literacy, attitudes, barriers, and perceived clinical utility among surgical healthcare learners and professionals in Jordan. AI exposure refers to participants’ prior interaction with or familiarity with AI technologies. AI literacy refers to the competency-related ability to understand, evaluate, and appropriately use AI tools. Confidence and attitudes represent participants’ perceptions and self-reported readiness to engage with AI technologies in clinical contexts.

The study was conducted across multiple teaching hospitals and academic medical centers in Jordan, including institutions with active surgical training programs. Data collection targeted environments where medical students, surgical residents, and practicing surgical specialists receive training or provide clinical care.

### 2.2 Conceptual relationship

AI exposure may act as an antecedent factor influencing AI literacy, which in turn may shape attitudes and confidence toward AI integration in clinical practice.

### 2.3 Population and sampling

Participants were eligible if they met any of the following categories: Medical students in clinical years (4th–6th year or equivalent), Surgical residents enrolled in accredited residency programs, or Surgical specialists (attendings/consultants) practicing in Jordan. Medical students were included as they represent future trainees and early adopters of emerging technologies, and their exposure to AI during medical education may influence its future integration into clinical and surgical practice. Although medical students have limited direct experience in surgical practice, their responses reflect perceptions and expectations regarding the potential role of AI in future clinical environments and surgical training.

### 2.4 Sample size

The minimum required sample size was calculated using Cochran’s formula for cross-sectional studies: *n* = Z2 *p * (1−p)/d2, Where: Z = 1.96 (95% confidence level), *p* = 0.5 (estimated prevalence), d = 0.05 (margin of error).

The calculation yielded a required sample size of 384 participants. Accounting for a 10% non-response rate, the final minimum required sample size was 423 participants.

Participants were recruited from three professional strata with unequal representation: medical students, residents, and consultants/specialists. To reflect the relative sizes of these groups and ensure adequate power for subgroup comparisons, a proportional allocation was applied: Medical students: 50%, Residents: 30%, Consultants/specialists: 20%. The higher proportion of medical students reflects their larger population size and accessibility within the study setting, as well as the study objective of capturing early exposure to AI technologies.

### 2.5 Participant identification and recruitment

Using stratified random sampling, participants were identified based on their professional role and availability during the study period:

1. Consultants/Specialists: Physicians with completed specialty training practicing in surgical or clinical departments.
2. Residents: Physicians currently enrolled in a residency program.
3. Medical Students: Undergraduate medical students in clinical years.

Eligible participants were contacted through institutional email lists and professional networks. Those who consented were included in the study, ensuring proportional representation across the three strata.

### 2.6 Instrumentation

A structured online questionnaire was developed and distributed using Google Forms, consisting of four major sections. Section A captured demographic and professional background information, including age, gender, level of study or years of training, surgical specialty, prior exposure to artificial intelligence, and previous experience with ChatGPT or other large language models. Section B assessed AI literacy using an adapted 18-item Meta-AI Literacy Scale, originally developed and validated by Carolus et al. (17), covering four domains: Use & Apply (Items 1–6), Know & Understand (Items 7–12), Detect AI (Items 13–15), and AI Ethics (Items 16–18). All items were rated on a 5-point Likert scale (1 = strongly disagree, 5 = strongly agree), and AI literacy was evaluated through domain-specific subscale averages and an overall composite score. Section C measured ChatGPT literacy, including frequency of use, understanding of its capabilities and limitations, perceived usefulness in medical education, research, and clinical tasks, and concerns related to accuracy, bias, confidentiality, and ethics; items were rated on a 5-point Likert scale, and a total score was calculated based on the sum of all items. Section D explored attitudes and barriers toward AI integration in surgical practice, assessing willingness to adopt AI in future clinical work, perceived need for institutional training or policies, perceived utility of AI in surgical and clinical settings, ethical and professional concerns, and understanding of legal and regulatory considerations. These sections were measured using items adapted from previously published literature (18–20).

### 2.7 Validation and piloting

The questionnaire was reviewed by a panel of five experts in surgical education, clinical AI, and survey methodology to ensure content validity, clarity, and relevance. Items were revised based on expert feedback. A pilot study was conducted with 20 participants representing each professional stratum (students, residents, and consultants) to evaluate readability, comprehension, and completion time. Minor adjustments were made to wording and instructions to improve clarity.

### 2.8 Subscale computation and reliability

Subscale scores were computed by averaging items within each domain, and an overall literacy score was calculated as the mean of all items. ChatGPT literacy and Attitudes Toward AI Integration were each measured using 8-item scales, with total scores representing the average of responses. Additionally, an AI Exposure subscale was included to assess participants’ self-reported understanding of key AI concepts, including Machine Learning (ML), Deep Learning (DL), Computer Vision (CV), Robotic Surgery AI, Clinical Decision Support AI, and Natural Language Processing (NLP). This exposure subscale demonstrated good internal consistency, with a Cronbach's *α* of 0.884. Item-level reliability analysis showed that Cronbach's *α* values would range from 0.848 to 0.887 if individual items were dropped, indicating that all items contributed meaningfully to the scale. All instruments demonstrated high internal consistency, with Cronbach's *α* values of 0.930 for the MAIL scale, 0.907 for the ChatGPT Literacy scale, and 0.846 for the Attitude scale. The combined questionnaire showed excellent reliability (*α* = 0.945), indicating strong internal coherence across all items and supporting the validity of the measures used.

### 2.9 Data collection and analysis

Data were collected electronically using Google Forms, allowing participants to complete the survey anonymously. The survey link was distributed via institutional email lists, professional networks, and surgical education platforms between 15/9/2025–30/11/2025. Participation was voluntary, and no incentives were offered.

All data were analyzed using R (version 4.5.1). Incomplete survey responses were excluded from the analysis to ensure data integrity, and no imputation was performed for missing values, as the proportion of missing data was minimal (<5%). Descriptive statistics were computed, including frequencies and percentages for categorical variables and means with standard deviations for continuous variables, along with summaries of overall AI literacy and ChatGPT literacy scores. For inferential analyses, assumptions for parametric tests were assessed prior to conducting ANOVA; normality was evaluated using the Shapiro–Wilk test, and homogeneity of variances was assessed with Levene's test. In cases where assumptions were violated, non-parametric alternatives (Kruskal–Wallis test) were applied. ANOVA or Kruskal–Wallis tests were used to compare AI literacy, ChatGPT literacy, attitudes, and barriers across student, resident, and specialist groups, followed by *post-hoc* pairwise comparisons using Tukey or Dwass-Steel-Critchlow-Fligner method tests as appropriate. Associations between categorical variables were examined using chi-square tests. Statistical significance was set at *p* < 0.05. Effect sizes were calculated to complement statistical significance and provide an estimate of the magnitude of observed differences. For Kruskal–Wallis tests, effect sizes were computed using epsilon squared (*ε*^2^). For chi-square tests, Cramer's V was used to assess the strength of associations. Effect sizes were reported alongside *p*-values to aid interpretation of practical and educational significance.

### 2.10 Ethical considerations

The study complies with the Declaration of Helsinki or relevant national research ethics guidelines. Ethical approval was obtained from the Institutional Review Board (IRB) of King Hussein Cancer Center (KHCC) prior to data collection (IRB number: 25 KHCC 256). Written informed consent was waived because the study involved minimal risk to participants and was conducted using an online survey, which did not collect any identifiable information. Participants provided electronic informed consent by agreeing to participate before accessing the survey, which ensured that they were fully informed about the study's purpose, procedures, and voluntary nature. This approach aligns with ethical guidelines for research involving minimal risk and remote data collection, where obtaining physical signatures is impractical and unnecessary for protecting participants’ rights and privacy.

## 3 Results

### 3.1 Participant demographics and AI exposure

**Figure 1:**
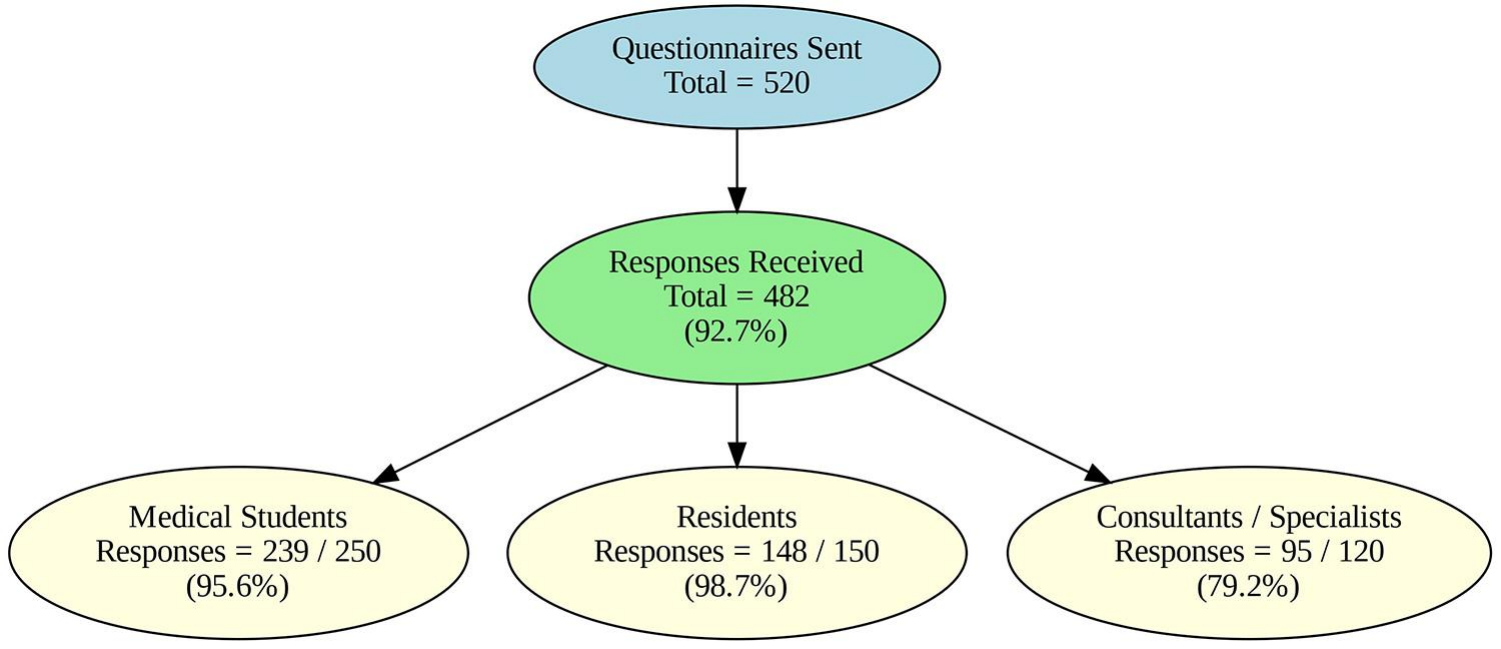
Response rates across professional groups.

A total of 520 questionnaires were distributed across three professional strata, and 482 were returned, yielding an overall response rate of 92.7%. Response rates by group were 95.6% for medical students (239/250), 98.7% for residents (148/150), and 79.2% for consultants/specialists (95/120) (Figure 1).

The sample demonstrated significant differences in demographic characteristics across groups. Consultants were older (mean 42.1 ± 9.4 years) compared with residents (26.8 ± 2.0 years) and students (22.4 ± 0.9 years; *p* < 0.001), and gender distribution varied significantly, with more females among students (71.1%) and residents (34.5%) than consultants (24.2%; *p* < 0.001). Most participants reported no formal AI training (93.2%), but a high proportion had used ChatGPT or similar AI tools for academic or clinical purposes (80.9%), with a moderate association between group and usage (*p* < 0.001, V = 0.3) (Table 1).

**Table 1:** Participant demographics and AI exposure .

Variable	Consultant/Specialist (*N* = 95)	Resident (*N* = 148)	Medical Student (*N* = 239)	Total (*N* = 482)	Effect Size^a^	*p*-value
**Age**					0.36	<0.001^1^
Mean (SD)	42.1 (9.4)	26.8 (2.0)	22.4 (0.9)	27.7 (8.6)		
Range	29–72	23–31	21–26	21–72		
**Gender**					0.46	<0.001^2^
Female	23 (24.2%)	51 (34.5%)	170 (71.1%)	244 (50.6%)		
Male	72 (75.8%)	97 (65.5%)	69 (28.9%)	238 (49.4%)		
**Formal training in AI**					0.06	0.377^2^
No	89 (93.7%)	141 (95.3%)	219 (91.6%)	449 (93.2%)		
Yes	6 (6.3%)	7 (4.7%)	20 (8.4%)	33 (6.8%)		
**Use of ChatGPT for medical/academic purposes**					0.3	<0.001^2^
No	38 (40.0%)	24 (16.2%)	30 (12.6%)	92 (19.1%)		
Yes	57 (60.0%)	124 (83.8%)	209 (87.4%)	390 (80.9%)		
**Understanding of AI Concepts**						
**Machine Learning (ML)**					0.10	0.312^2^
Never Heard	40 (42.1%)	56 (37.8%)	87 (36.4%)	183 (38.0%)		
Heard of It	35 (36.8%)	59 (39.9%)	75 (31.4%)	169 (35.1%)		
Understand Basics	16 (16.8%)	24 (16.2%)	53 (22.2%)	93 (19.3%)		
Understand Well	4 (4.2%)	6 (4.1%)	18 (7.5%)	28 (5.8%)		
Can Apply	0 (0.0%)	3 (2.0%)	6 (2.5%)	9 (1.9%)		
**Deep Learning (DL)**					0.11	0.164^2^
Never Heard	42 (44.2%)	72 (48.6%)	90 (37.7%)	204 (42.3%)		
Heard of It	38 (40.0%)	44 (29.7%)	82 (34.3%)	164 (34.0%)		
Understand Basics	11 (11.6%)	23 (15.5%)	48 (20.1%)	82 (17.0%)		
Understand Well	4 (4.2%)	8 (5.4%)	13 (5.4%)	25 (5.2%)		
Can Apply	0 (0.0%)	1 (0.7%)	6 (2.5%)	7 (1.5%)		
**Computer Vision (CV)**					0.2	0.021^2^
Never Heard	52 (54.7%)	74 (50.0%)	95 (39.7%)	221 (45.9%)		
Heard of It	29 (30.5%)	48 (32.4%)	69 (28.9%)	146 (30.3%)		
Understand Basics	11 (11.6%)	20 (13.5%)	51 (21.3%)	82 (17.0%)		
Understand Well	3 (3.2%)	5 (3.4%)	17 (7.1%)	25 (5.2%)		
Can Apply	0 (0.0%)	1 (0.7%)	7 (2.9%)	8 (1.7%)		
**Robotic Surgery AI**					0.3	0.008^2^
Never Heard	2 (2.1%)	24 (16.2%)	39 (16.3%)	65 (13.5%)		
Heard of It	47 (49.5%)	67 (45.3%)	112 (46.9%)	226 (46.9%)		
Understand Basics	38 (40.0%)	41 (27.7%)	62 (25.9%)	141 (29.3%)		
Understand Well	8 (8.4%)	15 (10.1%)	20 (8.4%)	43 (8.9%)		
Can Apply	0 (0.0%)	1 (0.7%)	6 (2.5%)	7 (1.5%)		
**Clinical Decision Support AI**					0.10	0.214^2^
Never Heard	33 (34.7%)	40 (27.0%)	55 (23.0%)	128 (26.6%)		
Heard of It	36 (37.9%)	56 (37.8%)	101 (42.3%)	193 (40.0%)		
Understand Basics	18 (18.9%)	40 (27.0%)	55 (23.0%)	113 (23.4%)		
Understand Well	8 (8.4%)	8 (5.4%)	18 (7.5%)	34 (7.1%)		
Can Apply	0 (0.0%)	4 (2.7%)	10 (4.2%)	14 (2.9%)		
**Natural Language Processing (NLP)**					0.12	0.081^2^
Never Heard	53 (55.8%)	86 (58.1%)	108 (45.2%)	247 (51.2%)		
Heard of It	28 (29.5%)	42 (28.4%)	71 (29.7%)	141 (29.3%)		
Understand Basics	12 (12.6%)	14 (9.5%)	40 (16.7%)	66 (13.7%)		
Understand Well	2 (2.1%)	5 (3.4%)	13 (5.4%)	20 (4.1%)		
Can Apply	0 (0.0%)	1 (0.7%)	7 (2.9%)	8 (1.7%)		
**AI Exposure Score**					0.1	0.020^1^
Mean (SD)	11.4 (3.9)	11.6 (4.0)	12.7 (5.1)	12.1 (4.6)		
Range	6–24	6–24	6–30	6–30		

*p*-values calculated using ^1^ANOVA/ Kruskal–Wallis test for continuous variables and ^2^Pearson's Chi-squared test for categorical variables.

a Effect sizes were calculated using *ε*^2^ for Kruskal–Wallis tests and Cramer's V for Chi-square tests.

Participants’ exposure to AI, measured by the exposure score, showed a small but statistically significant difference across groups (*p* = 0.020, *ε*^2^ = 0.10), with students showing the highest mean score (12.7 ± 5.1) compared with consultants (11.4 ± 3.9) and residents (11.6 ± 4.0). Self-reported understanding of specific AI concepts, including machine learning, deep learning, computer vision, robotic surgery AI, clinical decision support AI, and natural language processing, showed variable awareness. Significant differences were observed for computer vision (*p* = 0.021, V = 0.2) and robotic surgery AI (*p* = 0.008, V = 0.3). While consultants demonstrated the highest overall awareness of robotic surgery AI (with the lowest proportion reporting “never heard”), students and residents more frequently reported relatively higher-level familiarity categories such as “can apply” (Table 1).

### 3.2 Use & apply domain

Participants’ self-reported ability to use and apply AI tools differed across groups. Significant differences were observed in agreement levels for several items within this domain, including knowing how to use AI tools in medical or surgical settings (*p* < 0.001), having used AI applications to support clinical decision-making (*p* = 0.004), integrating AI tools into clinical practice (*p* = 0.025), critically assessing AI-based recommendations (*p* = 0.012), and actively engaging with AI tools for learning or practice (*p* < 0.001, V = 0.3). The strongest association was observed for active engagement with AI tools, indicating a moderate effect size, while other items demonstrated small effect sizes.

Agreement with the statement regarding identifying appropriate clinical scenarios for AI showed a trend toward significance (*p* = 0.052, V = 0.12), with a small effect size.

These differences reflect variation in the distribution of Likert-scale responses across groups rather than a comparison of specific response categories. The composite MAIL_Use&Apply score was significantly higher among residents (19.5 ± 5.2) compared with students (18.3 ± 5.6) and consultants (17.4 ± 5.4; *p* = 0.014, *ε*^2^ = 0.04). However, the magnitude of this difference was small, suggesting considerable overlap between groups in overall perceived ability to use and apply AI tools (Table 2).

**Table 2:** Use & apply domain of AI literacy - participant self-reported ability to apply AI tools in clinical and educational tasks.

Item	Consultant/Specialist (*N* = 95)	Resident (*N* = 148)	Medical Student (*N* = 239)	Total (*N* = 482)	Effect Size*	*p*-value
**I know how to use AI tools in surgical or medical settings**					0.24	<0.001^1^
Strongly disagree	7.0 (7.4%)	16.0 (10.8%)	50.0 (20.9%)	73.0 (15.1%)		
Disagree	30.0 (31.6%)	24.0 (16.2%)	64.0 (26.8%)	118.0 (24.5%)		
Neutral	24.0 (25.3%)	54.0 (36.5%)	68.0 (28.5%)	146.0 (30.3%)		
Agree	31.0 (32.6%)	45.0 (30.4%)	41.0 (17.2%)	117.0 (24.3%)		
Strongly agree	3.0 (3.2%)	9.0 (6.1%)	16.0 (6.7%)	28.0 (5.8%)		
**I have used AI applications to support clinical decision-making**					0.20	0.004^1^
Strongly disagree	12.0 (12.6%)	11.0 (7.4%)	40.0 (16.7%)	63.0 (13.1%)		
Disagree	22.0 (23.2%)	22.0 (14.9%)	43.0 (18.0%)	87.0 (18.0%)		
Neutral	27.0 (28.4%)	36.0 (24.3%)	70.0 (29.3%)	133.0 (27.6%)		
Agree	31.0 (32.6%)	67.0 (45.3%)	63.0 (26.4%)	161.0 (33.4%)		
Strongly agree	3.0 (3.2%)	12.0 (8.1%)	23.0 (9.6%)	38.0 (7.9%)		
**I feel confident in integrating AI tools in my clinical practice**					0.15	0.025^1^
Strongly disagree	8.0 (8.4%)	12.0 (8.1%)	30.0 (12.6%)	50.0 (10.4%)		
Disagree	27.0 (28.4%)	20.0 (13.5%)	40.0 (16.7%)	87.0 (18.0%)		
Neutral	28.0 (29.5%)	52.0 (35.1%)	73.0 (30.5%)	153.0 (31.7%)		
Agree	28.0 (29.5%)	56.0 (37.8%)	71.0 (29.7%)	155.0 (32.2%)		
Strongly agree	4.0 (4.2%)	8.0 (5.4%)	25.0 (10.5%)	37.0 (7.7%)		
**I can identify appropriate clinical scenarios where AI could be useful.**					0.12	0.052^1^
Strongly disagree	11.0 (11.6%)	10.0 (6.8%)	21.0 (8.8%)	42.0 (8.7%)		
Disagree	23.0 (24.2%)	17.0 (11.5%)	38.0 (15.9%)	78.0 (16.2%)		
Neutral	25.0 (26.3%)	39.0 (26.4%)	72.0 (30.1%)	136.0 (28.2%)		
Agree	31.0 (32.6%)	69.0 (46.6%)	81.0 (33.9%)	181.0 (37.6%)		
Strongly agree	5.0 (5.3%)	13.0 (8.8%)	27.0 (11.3%)	45.0 (9.3%)		
**I am able to critically assess AI-based recommendations.**					0.15	0.012^1^
Strongly disagree	8.0 (8.4%)	12.0 (8.1%)	29.0 (12.1%)	49.0 (10.2%)		
Disagree	27.0 (28.4%)	21.0 (14.2%)	41.0 (17.2%)	89.0 (18.5%)		
Neutral	22.0 (23.2%)	51.0 (34.5%)	83.0 (34.7%)	156.0 (32.4%)		
Agree	35.0 (36.8%)	54.0 (36.5%)	63.0 (26.4%)	152.0 (31.5%)		
Strongly agree	3.0 (3.2%)	10.0 (6.8%)	23.0 (9.6%)	36.0 (7.5%)		
**I have actively engaged with AI tools for learning or practice**					0.30	< 0.001^1^
Strongly disagree	14.0 (14.7%)	11.0 (7.4%)	27.0 (11.3%)	52.0 (10.8%)		
Disagree	30.0 (31.6%)	23.0 (15.5%)	23.0 (9.6%)	76.0 (15.8%)		
Neutral	23.0 (24.2%)	40.0 (27.0%)	61.0 (25.5%)	124.0 (25.7%)		
Agree	26.0 (27.4%)	57.0 (38.5%)	79.0 (33.1%)	162.0 (33.6%)		
Strongly agree	2.0 (2.1%)	17.0 (11.5%)	49.0 (20.5%)	68.0 (14.1%)		
**Summary Score (MAIL_Use&Apply)**					0.04	0.014^2^
Mean (SD)	17.4 (5.4)	19.5 (5.2)	18.3 (5.6)	18.5 (5.5)		
Range	6.0–30.0	6.0–30.0	6.0–30.0	6.0–30.0		

*p*-values calculated using ^1^ANOVA**/** Kruskal–Wallis test for continuous variables and ^2^Pearson's Chi-squared test for categorical variables.

*Effect sizes were calculated using ε² for Kruskal–Wallis tests and Cramer's V for Chi-square tests.

### 3.3 Know & understand domain

Self-reported understanding AI principles, types, limitations, and data quality effects showed moderate levels across all groups. Students reported relatively higher agreement with statements reflecting comprehension of AI functionality at a basic level (*p* = 0.043), whereas other items, including perceived knowledge of AI types in healthcare and distinguishing AI from automation or robotics, did not differ significantly. The overall MAIL_Know score was comparable across groups (mean ∼17.6 ± 5.1; *p* = 0.702) (Table 3).

**Table 3:** Know & understand domain of AI literacy – participant perceived knowledge and understanding of fundamental AI concepts and terminology.

Item	Consultant/Specialist (*N* = 95)	Resident (*N* = 148)	Medical Student (*N* = 239)	Total (*N* = 482)	Effect Size	*p*-value
**I understand how AI works at a basic level**					0.13	0.043^1^
Strongly disagree	5.0 (5.3%)	11.0 (7.4%)	23.0 (9.6%)	39.0 (8.1%)		
Disagree	22.0 (23.2%)	17.0 (11.5%)	40.0 (16.7%)	79.0 (16.4%)		
Neutral	36.0 (37.9%)	53.0 (35.8%)	70.0 (29.3%)	159.0 (33.0%)		
Agree	31.0 (32.6%)	56.0 (37.8%)	83.0 (34.7%)	170.0 (35.3%)		
Strongly agree	1.0 (1.1%)	11.0 (7.4%)	23.0 (9.6%)	35.0 (7.3%)		
**I am familiar with different types of AI used in healthcare**					0.10	0.463^1^
Strongly disagree	6.0 (6.3%)	13.0 (8.8%)	28.0 (11.7%)	47.0 (9.8%)		
Disagree	34.0 (35.8%)	46.0 (31.1%)	63.0 (26.4%)	143.0 (29.7%)		
Neutral	36.0 (37.9%)	49.0 (33.1%)	80.0 (33.5%)	165.0 (34.2%)		
Agree	18.0 (18.9%)	37.0 (25.0%)	60.0 (25.1%)	115.0 (23.9%)		
Strongly agree	1.0 (1.1%)	3.0 (2.0%)	8.0 (3.3%)	12.0 (2.5%)		
**I understand the limitations of AI systems**					0.11	0.144^1^
Strongly disagree	5.0 (5.3%)	12.0 (8.1%)	20.0 (8.4%)	37.0 (7.7%)		
Disagree	27.0 (28.4%)	28.0 (18.9%)	41.0 (17.2%)	96.0 (19.9%)		
Neutral	24.0 (25.3%)	47.0 (31.8%)	80.0 (33.5%)	151.0 (31.3%)		
Agree	36.0 (37.9%)	45.0 (30.4%)	76.0 (31.8%)	157.0 (32.6%)		
Strongly agree	3.0 (3.2%)	16.0 (10.8%)	22.0 (9.2%)	41.0 (8.5%)		
**I can explain how machine learning differs from traditional programming**					0.08	0.628^1^
Strongly disagree	11.0 (11.6%)	19.0 (12.8%)	40.0 (16.7%)	70.0 (14.5%)		
Disagree	30.0 (31.6%)	39.0 (26.4%)	65.0 (27.2%)	134.0 (27.8%)		
Neutral	30.0 (31.6%)	41.0 (27.7%)	71.0 (29.7%)	142.0 (29.5%)		
Agree	21.0 (22.1%)	43.0 (29.1%)	50.0 (20.9%)	114.0 (23.7%)		
Strongly agree	3.0 (3.2%)	6.0 (4.1%)	13.0 (5.4%)	22.0 (4.6%)		
**I can distinguish between AI, automation, and robotics in medicine**					0.11	0.170^1^
Strongly disagree	9.0 (9.5%)	30.0 (20.3%)	44.0 (18.4%)	83.0 (17.2%)		
Disagree	32.0 (33.7%)	34.0 (23.0%)	71.0 (29.7%)	137.0 (28.4%)		
Neutral	33.0 (34.7%)	53.0 (35.8%)	67.0 (28.0%)	153.0 (31.7%)		
Agree	17.0 (17.9%)	28.0 (18.9%)	44.0 (18.4%)	89.0 (18.5%)		
Strongly agree	4.0 (4.2%)	3.0 (2.0%)	13.0 (5.4%)	20.0 (4.1%)		
**I understand how data quality affects AI performance**					0.10	0.338^1^
Strongly disagree	8.0 (8.4%)	17.0 (11.5%)	29.0 (12.1%)	54.0 (11.2%)		
Disagree	26.0 (27.4%)	23.0 (15.5%)	51.0 (21.3%)	100.0 (20.7%)		
Neutral	21.0 (22.1%)	41.0 (27.7%)	58.0 (24.3%)	120.0 (24.9%)		
Agree	34.0 (35.8%)	50.0 (33.8%)	71.0 (29.7%)	155.0 (32.2%)		
Strongly agree	6.0 (6.3%)	17.0 (11.5%)	30.0 (12.6%)	53.0 (11.0%)		
**MAIL_Know (Overall** perceived **Knowledge Score)**					0.0006	0.702^2^
Mean (SD)	17.3 (4.9)	17.9 (5.0)	17.6 (5.2)	17.6 (5.1)		
Range	6.0–30.0	6.0–30.0	6.0–30.0	6.0–30.0		

*p*-values calculated using ^1^ANOVA**/** Kruskal–Wallis test for continuous variables and ^2^Pearson's Chi-squared test for categorical variables.

### 3.4 AI detect and ethics domains

The ability to detect AI use in clinical tools, differentiate AI-generated from human-generated content, and recognize AI-based decisions varied significantly across groups, but generally small-to-moderate differences across professional groups. Students and residents scored higher than consultants, reflected in a modest significant difference in the MAIL_Detect score (students 9.4 ± 3.0, residents 9.0 ± 2.7, consultants 8.5 ± 2.2; *p* = 0.033, *ε*^2^ = 0.12) (Table 4).

**Table 4:** Detect AI domain of AI literacy - participant ability to recognize AI-generated content or outputs in clinical and educational contexts.

Item	Consultant/Specialist (*N* = 95)	Resident (*N* = 148)	Medical Student (*N* = 239)	Total (*N* = 482)	Effect Size	*p*-value
**I can recognize when AI is being used in clinical tools**					0.20	0.003^1^
Strongly disagree	4.0 (4.2%)	12.0 (8.1%)	28.0 (11.7%)	44.0 (9.1%)		
Disagree	30.0 (31.6%)	27.0 (18.2%)	42.0 (17.6%)	99.0 (20.5%)		
Neutral	36.0 (37.9%)	64.0 (43.2%)	80.0 (33.5%)	180.0 (37.3%)		
Agree	24.0 (25.3%)	40.0 (27.0%)	68.0 (28.5%)	132.0 (27.4%)		
Strongly agree	1.0 (1.1%)	5.0 (3.4%)	21.0 (8.8%)	27.0 (5.6%)		
**I can differentiate between AI-generated and human-generated content**					0.27	< 0.001^1^
Strongly disagree	5.0 (5.3%)	13.0 (8.8%)	17.0 (7.1%)	35.0 (7.3%)		
Disagree	25.0 (26.3%)	27.0 (18.2%)	37.0 (15.5%)	89.0 (18.5%)		
Neutral	40.0 (42.1%)	50.0 (33.8%)	63.0 (26.4%)	153.0 (31.7%)		
Agree	24.0 (25.3%)	49.0 (33.1%)	89.0 (37.2%)	162.0 (33.6%)		
Strongly agree	1.0 (1.1%)	9.0 (6.1%)	33.0 (13.8%)	43.0 (8.9%)		
**I know when a decision is based on AI algorithms**					0.20	0.003^1^
Strongly disagree	4.0 (4.2%)	16.0 (10.8%)	26.0 (10.9%)	46.0 (9.5%)		
Disagree	36.0 (37.9%)	31.0 (20.9%)	57.0 (23.8%)	124.0 (25.7%)		
Neutral	38.0 (40.0%)	59.0 (39.9%)	77.0 (32.2%)	174.0 (36.1%)		
Agree	16.0 (16.8%)	37.0 (25.0%)	58.0 (24.3%)	111.0 (23.0%)		
Strongly agree	1.0 (1.1%)	5.0 (3.4%)	21.0 (8.8%)	27.0 (5.6%)		
**MAIL_Detect (Overall Detect AI Score)**					0.12	0.033^2^
Mean (SD)	8.5 (2.2)	9.0 (2.7)	9.4 (3.0)	9.1 (2.8)		
Range	3.0–15.0	3.0–15.0	3.0–15.0	3.0–15.0		

*p*-values calculated using ^1^ANOVA**/** Kruskal–Wallis test for continuous variables and ^2^Pearson's Chi-squared test for categorical variables.

Awareness of ethical considerations, data privacy, and the importance of guidelines in AI implementation was generally high across all groups, with no significant differences in the MAIL_AiEthics score (mean ∼10.8 ± 2.9; *p* = 0.786, *ε*^2^ = 0.0006) (Table 5).

**Table 5:** AI Ethics domain of AI literacy - participant awareness of ethical, legal, and safety considerations in the use of AI.

Item	Consultant/Specialist (*N* = 95)	Resident (*N* = 148)	Medical Student (*N* = 239)	Total (*N* = 482)	Effect Size	*p*-value
**I am aware of ethical concerns related to using AI in surgery**					0.12	0.068^1^
Strongly disagree	7.0 (7.4%)	9.0 (6.1%)	26.0 (10.9%)	42.0 (8.7%)		
Disagree	13.0 (13.7%)	25.0 (16.9%)	35.0 (14.6%)	73.0 (15.1%)		
Neutral	17.0 (17.9%)	42.0 (28.4%)	63.0 (26.4%)	122.0 (25.3%)		
Agree	49.0 (51.6%)	51.0 (34.5%)	78.0 (32.6%)	178.0 (36.9%)		
Strongly agree	9.0 (9.5%)	21.0 (14.2%)	37.0 (15.5%)	67.0 (13.9%)		
**I understand the importance of data privacy and confidentiality in AI applications**					0.11	0.099^1^
Strongly disagree	2.0 (2.1%)	5.0 (3.4%)	17.0 (7.1%)	24.0 (5.0%)		
Disagree	8.0 (8.4%)	12.0 (8.1%)	22.0 (9.2%)	42.0 (8.7%)		
Neutral	22.0 (23.2%)	40.0 (27.0%)	48.0 (20.1%)	110.0 (22.8%)		
Agree	50.0 (52.6%)	63.0 (42.6%)	94.0 (39.3%)	207.0 (42.9%)		
Strongly agree	13.0 (13.7%)	28.0 (18.9%)	58.0 (24.3%)	99.0 (20.5%)		
**I believe ethical guidelines should be mandatory in AI tool implementation**					0.13	0.059^1^
Strongly disagree	5.0 (5.3%)	7.0 (4.7%)	14.0 (5.9%)	26.0 (5.4%)		
Disagree	6.0 (6.3%)	12.0 (8.1%)	11.0 (4.6%)	29.0 (6.0%)		
Neutral	12.0 (12.6%)	39.0 (26.4%)	43.0 (18.0%)	94.0 (19.5%)		
Agree	46.0 (48.4%)	50.0 (33.8%)	85.0 (35.6%)	181.0 (37.6%)		
Strongly agree	26.0 (27.4%)	40.0 (27.0%)	86.0 (36.0%)	152.0 (31.5%)		
**MAIL_AI_ethics (Overall AI Ethics Score)**					0.0006	0.786^2^
Mean (SD)	11.0 (2.7)	10.7 (2.8)	10.8 (3.1)	10.8 (2.9)		
Range	3.0–15.0	3.0–15.0	3.0–15.0	3.0–15.0		

*p*-values calculated using ^1^ANOVA**/** Kruskal–Wallis test for continuous variables and ^2^Pearson's Chi-squared test for categorical variables.

### 3.5 AI literacy overall_score

**Figure 2:**
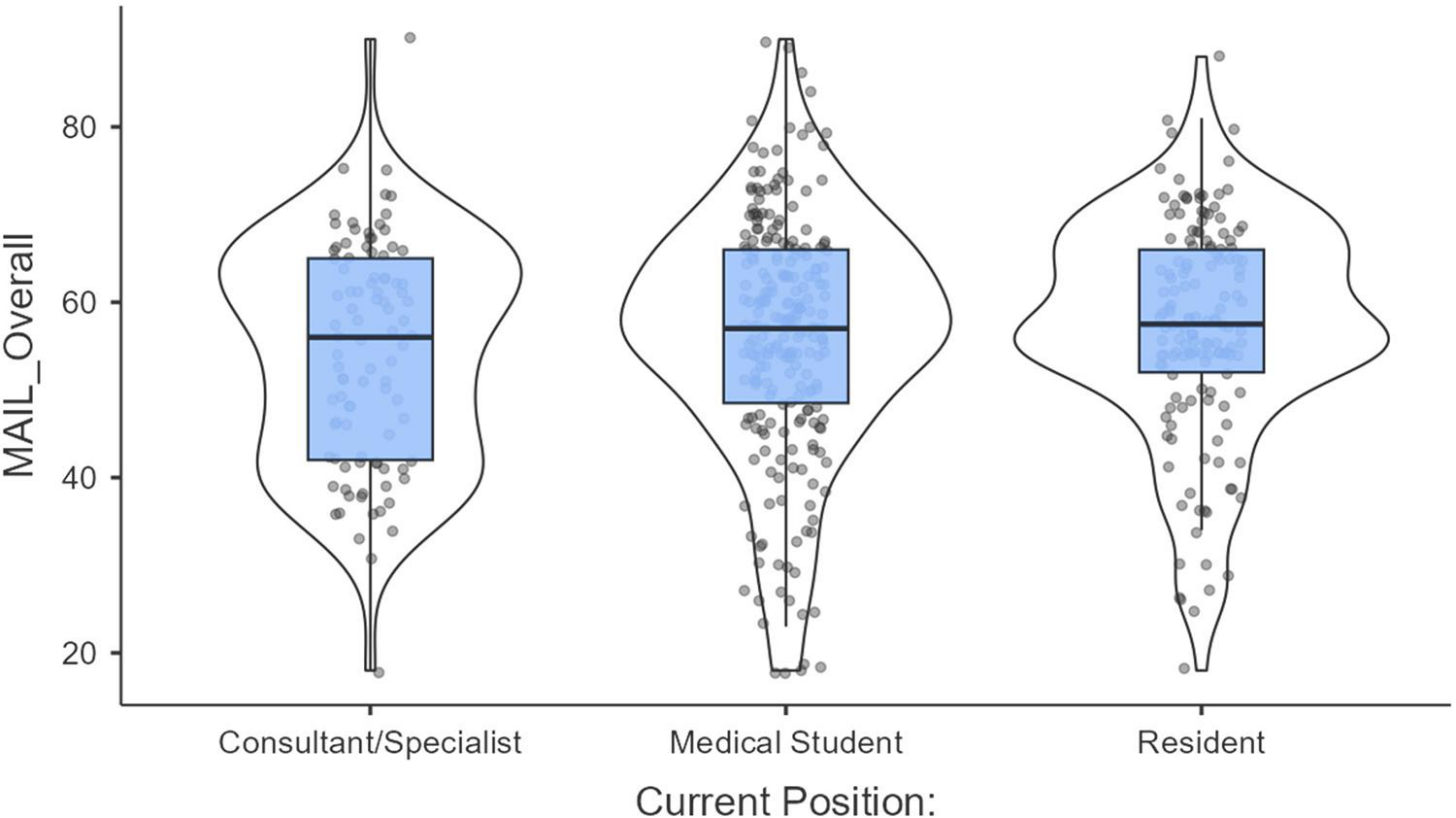
Distribution of meta-AI literacy (MAIL) scores across professional groups. Box-and-violin plots illustrate the distribution of MAIL scores among medical students, residents, and consultants/specialists.

The overall Meta-AI Literacy (MAIL) scores across professional groups reported moderate to high AI literacy among participants. Medical students had the highest median MAIL scores, followed closely by residents, while consultants/specialists showed slightly lower median scores. The distribution of scores was relatively wide within each group, indicating variability in AI literacy at the individual level, with some outliers observed across all groups. A Kruskal–Wallis test was conducted to assess differences in MAIL scores across professional groups, revealing no statistically significant differences (*χ*^2^ = 3.06, df = 2, *p* = 0.217, *ε*^2^ = 0.00635). Pairwise comparisons using the Dwass-Steel-Critchlow-Fligner method similarly showed no significant differences between consultants/specialists and medical students (W = 1.901, *p* = 0.371), consultants/specialists and residents (W = 2.493, *p* = 0.182), or medical students and residents (W = 0.766, *p* = 0.851). These findings suggest that overall score of AI literacy is comparable across medical students, residents, and consultants/specialists, although individual variability highlights the potential for targeted educational initiatives (Figure 2).

### 3.6 ChatGPT literacy

Participants’ familiarity and agreement with ChatGPT differed across professional groups. Medical students and residents reported more frequent use of ChatGPT and greater awareness of its capabilities, limitations, and usefulness for educational or clinical purposes, particularly for frequent use in academic or clinical contexts (*p* < 0.001, V = 0.35) and understanding of task suitability (*p* < 0.001, V = 0.30), both demonstrating moderate-to-strong effect sizes. Agreement in evaluating ChatGPT outputs was higher among students and residents than consultants (*p* = 0.025), while self-reported understanding of ethical considerations in ChatGPT use did not differ significantly. Overall, the mean ChatGPT literacy score was highest among students (29.8 ± 6.4), followed by residents (29.2 ± 6.0) and consultants (26.5 ± 6.3; *p* < 0.001, *ε*^2^ = 0.17), reflecting a moderate effect size and suggesting greater familiarity and engagement with AI tools among younger participants (Table 6).

**Table 6:** ChatGPT literacy among consultants, residents, and medical students (*N* = 482).

Item	Consultant/Specialist (*N* = 95)	Resident (*N* = 148)	Medical Student (*N* = 239)	Total (*N* = 482)	Effect Size	*p* value
**I frequently use ChatGPT (or similar tools) in my studies/work.**					0.35	<0.001^1^
Strongly disagree	5.0 (5.3%)	6.0 (4.1%)	13.0 (5.4%)	24.0 (5.0%)		
Disagree	27.0 (28.4%)	11.0 (7.4%)	5.0 (2.1%)	43.0 (8.9%)		
Neutral	21.0 (22.1%)	27.0 (18.2%)	40.0 (16.7%)	88.0 (18.3%)		
Agree	36.0 (37.9%)	68.0 (45.9%)	86.0 (36.0%)	190.0 (39.4%)		
Strongly agree	6.0 (6.3%)	36.0 (24.3%)	95.0 (39.7%)	137.0 (28.4%)		
**I understand the types of tasks ChatGPT is good at**					0.3	<0.001^1^
Strongly disagree	6.0 (6.3%)	4.0 (2.7%)	9.0 (3.8%)	19.0 (3.9%)		
Disagree	27.0 (28.4%)	15.0 (10.1%)	15.0 (6.3%)	57.0 (11.8%)		
Neutral	27.0 (28.4%)	43.0 (29.1%)	58.0 (24.3%)	128.0 (26.6%)		
Agree	34.0 (35.8%)	63.0 (42.6%)	88.0 (36.8%)	185.0 (38.4%)		
Strongly agree	1.0 (1.1%)	23.0 (15.5%)	69.0 (28.9%)	93.0 (19.3%)		
**I am aware of the limitations and potential errors of ChatGPT**					0.20	<0.001^1^
Strongly disagree	3.0 (3.2%)	3.0 (2.0%)	9.0 (3.8%)	15.0 (3.1%)		
Disagree	24.0 (25.3%)	13.0 (8.8%)	20.0 (8.4%)	57.0 (11.8%)		
Neutral	19.0 (20.0%)	36.0 (24.3%)	55.0 (23.0%)	110.0 (22.8%)		
Agree	43.0 (45.3%)	68.0 (45.9%)	99.0 (41.4%)	210.0 (43.6%)		
Strongly agree	6.0 (6.3%)	28.0 (18.9%)	56.0 (23.4%)	90.0 (18.7%)		
**I believe ChatGPT is useful for clinical or educational purposes**					0.21	<0.001^1^
Strongly disagree	3.0 (3.2%)	6.0 (4.1%)	8.0 (3.3%)	17.0 (3.5%)		
Disagree	19.0 (20.0%)	7.0 (4.7%)	10.0 (4.2%)	36.0 (7.5%)		
Neutral	18.0 (18.9%)	26.0 (17.6%)	42.0 (17.6%)	86.0 (17.8%)		
Agree	48.0 (50.5%)	76.0 (51.4%)	110.0 (46.0%)	234.0 (48.5%)		
Strongly agree	7.0 (7.4%)	33.0 (22.3%)	69.0 (28.9%)	109.0 (22.6%)		
**I am concerned about the reliability of ChatGPT outputs in a clinical context**					0.15	0.024^1^
Strongly disagree	3.0 (3.2%)	4.0 (2.7%)	11.0 (4.6%)	18.0 (3.7%)		
Disagree	16.0 (16.8%)	15.0 (10.1%)	17.0 (7.1%)	48.0 (10.0%)		
Neutral	17.0 (17.9%)	41.0 (27.7%)	63.0 (26.4%)	121.0 (25.1%)		
Agree	49.0 (51.6%)	60.0 (40.5%)	94.0 (39.3%)	203.0 (42.1%)		
Strongly agree	10.0 (10.5%)	28.0 (18.9%)	54.0 (22.6%)	92.0 (19.1%)		
**I believe ChatGPT can enhance surgical education**					0.10	0.339^1^
Strongly disagree	1.0 (1.1%)	4.0 (2.7%)	12.0 (5.0%)	17.0 (3.5%)		
Disagree	11.0 (11.6%)	14.0 (9.5%)	25.0 (10.5%)	50.0 (10.4%)		
Neutral	25.0 (26.3%)	36.0 (24.3%)	69.0 (28.9%)	130.0 (27.0%)		
Agree	47.0 (49.5%)	68.0 (45.9%)	90.0 (37.7%)	205.0 (42.5%)		
Strongly agree	11.0 (11.6%)	26.0 (17.6%)	43.0 (18.0%)	80.0 (16.6%)		
**I feel confident evaluating the quality of ChatGPT's responses**					0.15	0.025^1^
Strongly disagree	5.0 (5.3%)	6.0 (4.1%)	9.0 (3.8%)	20.0 (4.1%)		
Disagree	18.0 (18.9%)	23.0 (15.5%)	25.0 (10.5%)	66.0 (13.7%)		
Neutral	28.0 (29.5%)	41.0 (27.7%)	82.0 (34.3%)	151.0 (31.3%)		
Agree	41.0 (43.2%)	62.0 (41.9%)	84.0 (35.1%)	187.0 (38.8%)		
Strongly agree	3.0 (3.2%)	16.0 (10.8%)	39.0 (16.3%)	58.0 (12.0%)		
**I understand the ethical considerations of using ChatGPT in clinical settings**					0.11	0.190^1^
Strongly disagree	2.0 (2.1%)	4.0 (2.7%)	10.0 (4.2%)	16.0 (3.3%)		
Disagree	16.0 (16.8%)	14.0 (9.5%)	22.0 (9.2%)	52.0 (10.8%)		
Neutral	22.0 (23.2%)	40.0 (27.0%)	61.0 (25.5%)	123.0 (25.5%)		
Agree	46.0 (48.4%)	67.0 (45.3%)	98.0 (41.0%)	211.0 (43.8%)		
Strongly agree	9.0 (9.5%)	23.0 (15.5%)	48.0 (20.1%)	80.0 (16.6%)		
**ChatGPT Literacy total score**					0.17	<0.001^2^
Mean (SD)	26.5 (6.3)	29.2 (6.0)	29.8 (6.4)	29.0 (6.4)		
Range	9.0–40.0	8.0–40.0	8.0–40.0	8.0–40.0		

*p*-values calculated using ^1^ANOVA/ Kruskal–Wallis test for continuous variables and ^2^Pearson's Chi-squared test for categorical variables.

### 3.7 Attitudes toward AI integration into the clinical practice

Participants generally expressed positive attitudes toward integrating AI into clinical practice, with statistically significant but modest differences across professional groups. Willingness to integrate AI varied significantly (*p* = 0.004, V = 0.26), with residents demonstrating the highest agreement, indicating a moderate effect size. Concerns regarding potential legal or regulatory risks also varied significantly (*p* = 0.010), with consultants expressing greater concern compared with students and residents. However, most remaining attitude items showed small or negligible effect sizes.

Importantly, a majority of participants across all groups agreed or strongly agreed that the use of AI in clinical practice could potentially lead to physician over-reliance or deskilling (consultants 58.9%, residents 62.2%, medical students 51.8%). Participants also widely agreed that AI literacy should be incorporated into surgical education, and many expressed interests in attending AI-related training programs, although these differences were not statistically significant. The mean Attitudes and Barriers score was comparable across groups (27.7–28.4; *p* = 0.476), indicating generally positive attitudes toward AI integration and training (Table 7).

**Table 7:** Attitudes toward AI integration in clinical practice among consultants, medical students, and residents.

Attitude item	Consultant/Specialist (*N* = 95)	Resident (*N* = 148)	Medical Student (*N* = 239)	Total (*N* = 482)	Effect Size	*p* value
I am willing to integrate AI into my clinical practice					0.26	0.004^1^
**Strongly disagree**	3.0 (3.2%)	6.0 (4.1%)	15.0 (6.3%)	24.0 (5.0%)		
**Disagree**	11.0 (11.6%)	3.0 (2.0%)	20.0 (8.4%)	34.0 (7.1%)		
**Neutral**	37.0 (38.9%)	46.0 (31.1%)	93.0 (38.9%)	176.0 (36.5%)		
**Agree**	40.0 (42.1%)	71.0 (48.0%)	81.0 (33.9%)	192.0 (39.8%)		
**Strongly agree**	4.0 (4.2%)	22.0 (14.9%)	30.0 (12.6%)	56.0 (11.6%)		
My institution supports AI training or use in clinical settings					0.12	0.090^1^
**Strongly disagree**	12.0 (12.6%)	29.0 (19.6%)	23.0 (9.6%)	64.0 (13.3%)		
**Disagree**	33.0 (34.7%)	44.0 (29.7%)	70.0 (29.3%)	147.0 (30.5%)		
**Neutral**	32.0 (33.7%)	44.0 (29.7%)	80.0 (33.5%)	156.0 (32.4%)		
**Agree**	14.0 (14.7%)	20.0 (13.5%)	53.0 (22.2%)	87.0 (18.0%)		
**Strongly agree**	4.0 (4.2%)	11.0 (7.4%)	13.0 (5.4%)	28.0 (5.8%)		
I feel that I need more training to confidently use AI tools					0.05	0.964^1^
**Strongly disagree**	2.0 (2.1%)	4.0 (2.7%)	8.0 (3.3%)	14.0 (2.9%)		
**Disagree**	9.0 (9.5%)	9.0 (6.1%)	20.0 (8.4%)	38.0 (7.9%)		
**Neutral**	16.0 (16.8%)	33.0 (22.3%)	50.0 (20.9%)	99.0 (20.5%)		
**Agree**	43.0 (45.3%)	64.0 (43.2%)	100.0 (41.8%)	207.0 (42.9%)		
**Strongly agree**	25.0 (26.3%)	38.0 (25.7%)	61.0 (25.5%)	124.0 (25.7%)		
Attitudes and Barriers [I am concerned about legal or regulatory risks when using AI					0.20	0.010^1^
**Strongly disagree**	4.0 (4.2%)	1.0 (0.7%)	10.0 (4.2%)	15.0 (3.1%)		
**Disagree**	8.0 (8.4%)	8.0 (5.4%)	24.0 (10.0%)	40.0 (8.3%)		
**Neutral**	19.0 (20.0%)	52.0 (35.1%)	72.0 (30.1%)	143.0 (29.7%)		
**Agree**	53.0 (55.8%)	64.0 (43.2%)	86.0 (36.0%)	203.0 (42.1%)		
**Strongly agree**	11.0 (11.6%)	23.0 (15.5%)	47.0 (19.7%)	81.0 (16.8%)		
I believe AI can improve surgical outcomes					0.08	0.588^1^
**Strongly disagree**	3.0 (3.2%)	5.0 (3.4%)	11.0 (4.6%)	19.0 (3.9%)		
**Disagree**	6.0 (6.3%)	9.0 (6.1%)	23.0 (9.6%)	38.0 (7.9%)		
**Neutral**	31.0 (32.6%)	40.0 (27.0%)	66.0 (27.6%)	137.0 (28.4%)		
**Agree**	46.0 (48.4%)	67.0 (45.3%)	103.0 (43.1%)	216.0 (44.8%)		
**Strongly agree**	9.0 (9.5%)	27.0 (18.2%)	36.0 (15.1%)	72.0 (14.9%)		
I worry that AI could lead to over-reliance or deskilling of physicians					0.15	0.006^1^
**Strongly disagree**	4.0 (4.2%)	3.0 (2.0%)	13.0 (5.4%)	20.0 (4.1%)		
**Disagree**	12.0 (12.6%)	8.0 (5.4%)	34.0 (14.2%)	54.0 (11.2%)		
**Neutral**	23.0 (24.2%)	45.0 (30.4%)	68.0 (28.5%)	136.0 (28.2%)		
**Agree**	40.0 (42.1%)	67.0 (45.3%)	67.0 (28.0%)	174.0 (36.1%)		
**Strongly agree**	16.0 (16.8%)	25.0 (16.9%)	57.0 (23.8%)	98.0 (20.3%)		
I think AI literacy should be a core part of surgical education					0.11	0.118^1^
**Strongly disagree**	3.0 (3.2%)	5.0 (3.4%)	10.0 (4.2%)	18.0 (3.7%)		
**Disagree**	6.0 (6.3%)	14.0 (9.5%)	30.0 (12.6%)	50.0 (10.4%)		
**Neutral**	34.0 (35.8%)	52.0 (35.1%)	79.0 (33.1%)	165.0 (34.2%)		
**Agree**	46.0 (48.4%)	62.0 (41.9%)	82.0 (34.3%)	190.0 (39.4%)		
**Strongly agree**	6.0 (6.3%)	15.0 (10.1%)	38.0 (15.9%)	59.0 (12.2%)		
I am interested in attending AI-related training sessions or workshops					0.08	0.610^1^
**Strongly disagree**	4.0 (4.2%)	5.0 (3.4%)	13.0 (5.4%)	22.0 (4.6%)		
**Disagree**	7.0 (7.4%)	11.0 (7.4%)	22.0 (9.2%)	40.0 (8.3%)		
**Neutral**	21.0 (22.1%)	33.0 (22.3%)	58.0 (24.3%)	112.0 (23.2%)		
**Agree**	46.0 (48.4%)	62.0 (41.9%)	85.0 (35.6%)	193.0 (40.0%)		
**Strongly agree**	17.0 (17.9%)	37.0 (25.0%)	61.0 (25.5%)	115.0 (23.9%)		
Sum Score – Attitude Toward AI Integration					0.001	0.476^2^
** Mean (SD)**	27.7 (5.5)	28.4 (5.0)	27.7 (6.0)	27.9 (5.6)		
** Range**	8.0–40.0	12.0–40.0	8.0–40.0	8.0–40.0		

*p*-values calculated using ^1^ANOVA/ Kruskal–Wallis test for continuous variables and ^2^Pearson's Chi-squared test for categorical variables.

### 3.8 Perceived AI utility in clinical domains

Participants generally perceived AI as useful across multiple surgical and clinical areas, with residents and medical students reporting higher perceived utility than consultants. AI was seen as particularly helpful for surgical planning, diagnostic accuracy, and robotic-assisted surgery. Consultants were more cautious regarding intraoperative decision-making, while all groups viewed AI as moderately to highly useful for medical documentation. Statistically significant differences between groups were observed for surgical planning (*p* = 0.003), diagnostic accuracy (*p* = 0.001), intraoperative decision-making (*p* < 0.001), and surgical robotics (*p* = 0.007), whereas differences in medical documentation were marginal (*p* = 0.051) (Table 8).

**Table 8:** Perceived utility of AI in surgical and clinical practice among consultants, residents, and medical students.

How do you perceive AI in the following areas?	Consultant/Specialist (*N* = 95)	Resident (*N* = 148)	Medical Student (*N* = 239)	Total (*N* = 482)	Effect Size	*p* value
**Surgical planning**					0.20	0.003^1^
Not at all useful	8.0 (8.4%)	11.0 (7.4%)	24.0 (10.0%)	43.0 (8.9%)		
Useful	17.0 (17.9%)	39.0 (26.4%)	63.0 (26.4%)	119.0 (24.7%)		
Slightly useful	44.0 (46.3%)	30.0 (20.3%)	63.0 (26.4%)	137.0 (28.4%)		
Moderately useful	22.0 (23.2%)	54.0 (36.5%)	72.0 (30.1%)	148.0 (30.7%)		
Very useful	4.0 (4.2%)	14.0 (9.5%)	17.0 (7.1%)	35.0 (7.3%)		
**Diagnostic accuracy**					0.20	0.001^1^
Not at all useful	4.0 (4.2%)	5.0 (3.4%)	16.0 (6.7%)	25.0 (5.2%)		
Useful	15.0 (15.8%)	34.0 (23.0%)	72.0 (30.1%)	121.0 (25.1%)		
Slightly useful	39.0 (41.1%)	32.0 (21.6%)	44.0 (18.4%)	115.0 (23.9%)		
Moderately useful	32.0 (33.7%)	67.0 (45.3%)	90.0 (37.7%)	189.0 (39.2%)		
Very useful	5.0 (5.3%)	10.0 (6.8%)	17.0 (7.1%)	32.0 (6.6%)		
**Intraoperative decision-making**					0.25	< 0.001^1^
Not at all useful	36.0 (37.9%)	31.0 (20.9%)	37.0 (15.5%)	104.0 (21.6%)		
Useful	7.0 (7.4%)	16.0 (10.8%)	55.0 (23.0%)	78.0 (16.2%)		
Slightly useful	30.0 (31.6%)	52.0 (35.1%)	63.0 (26.4%)	145.0 (30.1%)		
Moderately useful	18.0 (18.9%)	43.0 (29.1%)	72.0 (30.1%)	133.0 (27.6%)		
Very useful	4.0 (4.2%)	6.0 (4.1%)	12.0 (5.0%)	22.0 (4.6%)		
**Surgical robotics**					0.15	0.007^1^
Not at all useful	9.0 (9.5%)	4.0 (2.7%)	22.0 (9.2%)	35.0 (7.3%)		
Useful	17.0 (17.9%)	45.0 (30.4%)	63.0 (26.4%)	125.0 (25.9%)		
Slightly useful	32.0 (33.7%)	24.0 (16.2%)	55.0 (23.0%)	111.0 (23.0%)		
Moderately useful	29.0 (30.5%)	52.0 (35.1%)	68.0 (28.5%)	149.0 (30.9%)		
Very useful	8.0 (8.4%)	23.0 (15.5%)	31.0 (13.0%)	62.0 (12.9%)		
**Medical documentation**					0.13	0.051^1^
Not at all useful	4.0 (4.2%)	1.0 (0.7%)	12.0 (5.0%)	17.0 (3.5%)		
Useful	25.0 (26.3%)	42.0 (28.4%)	75.0 (31.4%)	142.0 (29.5%)		
Slightly useful	15.0 (15.8%)	11.0 (7.4%)	26.0 (10.9%)	52.0 (10.8%)		
Moderately useful	30.0 (31.6%)	41.0 (27.7%)	54.0 (22.6%)	125.0 (25.9%)		
Very useful	21.0 (22.1%)	53.0 (35.8%)	72.0 (30.1%)	146.0 (30.3%)		

*p*-values calculated using ^1^Pearson's Chi-squared test for categorical variables.

### 3.9 Barrier and ethical concerns

**Figure 3:**
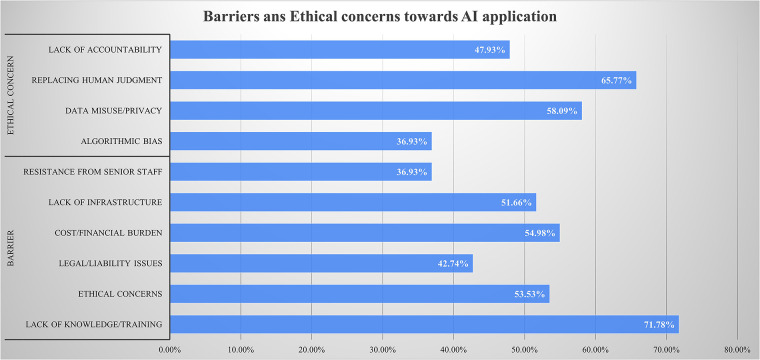
Barriers and ethical concerns regarding AI integration in clinical practice.

Participants identified several barriers and ethical concerns regarding AI integration in clinical practice. The most commonly reported barriers were lack of knowledge or training (71.8%), cost (55.0%), ethical concerns (53.5%), and lack of infrastructure (51.7%). Key ethical concerns included the potential for AI to replace human judgment (65.8%), risks of data misuse or privacy breaches (58.1%), lack of accountability (47.9%), and algorithmic bias (36.9%) (Figure 3).

### 3.10 Associations among AI literacy, ChatGPT literacy, and attitudes across professional groups

**Figure 4:**
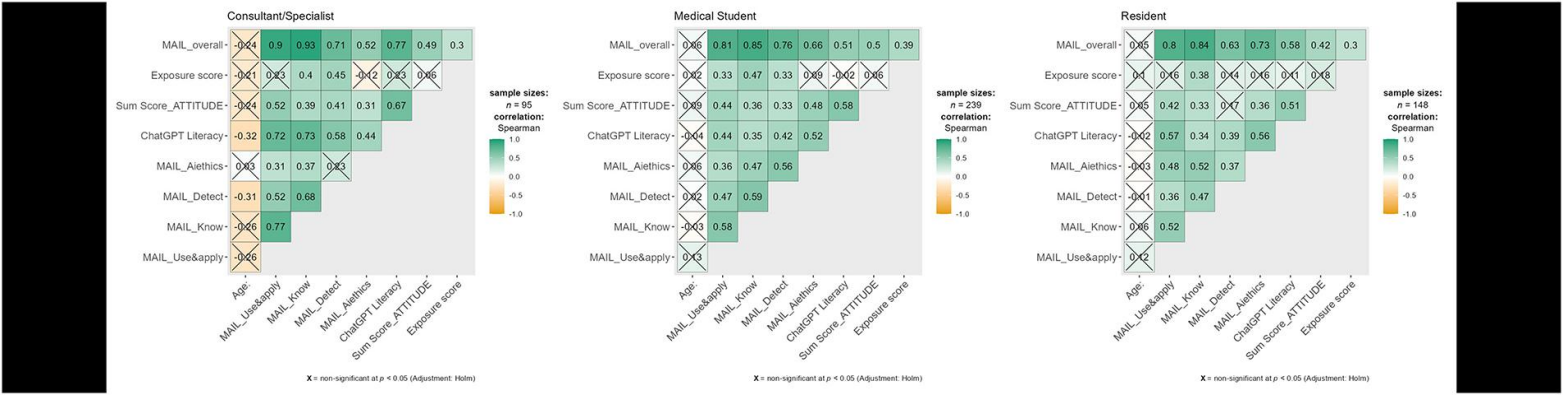
Spearman correlation matrices of AI/ChatGPT literacy measures across professional groups. Non-significant correlations after Holm adjustment are marked with an “X.” Color intensity represents correlation strength, with green indicating positive and orange indicating negative correlations.

Spearman correlation analyses examined relationships among AI literacy measures across consultants/specialists, medical students, and residents. As expected, MAIL_overall, a composite of MAIL_Use&Apply, MAIL_Know, MAIL_AI_Detect, and MAIL_AI_ethics, showed strong positive correlations with its constituent subdomains across all groups, confirming internal consistency. Beyond this, MAIL_overall was moderately associated with ChatGPT Literacy (consultants: r = 0.77; students: r = 0.51; residents: r = 0.58) and Attitude_score (consultants: r = 0.49; students: r = 0.50), indicating that higher AI literacy relates to better ChatGPT perceived knowledge and more positive attitudes toward AI. Among consultants, age was negatively correlated with MAIL subdomains and ChatGPT Literacy (ranging from −0.26 to −0.32), whereas age showed weaker or non-significant associations in students and residents. Correlations among MAIL subdomains and ChatGPT Literacy were generally moderate to strong across all groups, highlighting consistent relationships between AI perceived knowledge, ethical understanding, detection skills, and attitudes (Figure 4).

## 4 Discussion

This study assessed the perceived knowledge, attitudes, perceived usefulness, and barriers related to artificial intelligence (AI) and ChatGPT among medical students, residents, and consultants within a surgical setting. Overall, participants expressed generally positive attitudes toward AI integration, particularly regarding its potential to enhance diagnostic accuracy, surgical planning, documentation efficiency, and robotic-assisted surgery. However, willingness to adopt AI varied across professional groups, with medical students and residents tended to report greater openness, familiarity, and agreement in using AI tools, whereas consultants tended to be more cautious, particularly in domains involving intraoperative decision-making. Despite these differences, most participants acknowledged the value of AI and recognized the need for additional training to support its safe and effective integration into clinical workflows. Although several comparisons were statistically significant, effect sizes were generally small, suggesting substantial overlap between groups and limited practical separation in measured constructs.

The variations between trainees and consultants observed in this study mirror trends reported in the wider literature. Parrey et al. found that medical students overwhelmingly endorsed AI's benefits for improving access to clinical information and reducing errors, reflecting similar enthusiasm among trainees in our cohort (21). Likewise, the Taibah University study reported that although students recognized AI's transformative potential in medical education, their practical understanding of machine learning concepts and system limitations was limited—an observation consistent with our findings of moderate AI perceived knowledge across all groups (22). However, unlike the Taibah cohort, which focused primarily on educational applications, our study identified differences in self-reported understanding of clinically oriented AI applications, such as intraoperative decision-making and surgical robotics, likely reflecting differences in clinical exposure across professional levels.

Interestingly, students and residents reported relatively higher scores in the AI detection domain compared with consultants. One possible explanation is that less experienced learners may perceive tasks such as identifying AI-generated outputs or recognizing AI-assisted decisions as relatively straightforward because they have had limited exposure to real-world clinical AI systems. In contrast, consultants with greater clinical experience may have a more realistic appreciation of the complexity and limitations involved in identifying AI-generated content in practice (23).

Differences between participant groups may not solely reflect generational attitudes toward technology. Less experienced participants may have limited exposure to real-world AI applications in clinical settings, which could influence their perceptions of the technology's capabilities and challenges. In contrast, consultants may adopt a more cautious perspective based on greater clinical responsibility and exposure to practical implementation challenges. This pattern may reflect a form of overestimation among less experienced participants, a phenomenon previously described in educational research whereby individuals with limited experience may underestimate the difficulty of complex tasks (24).

Perceptions of ChatGPT also aligned with previous evidence. Tangadulrat et al. reported that medical students were significantly more positive than practicing physicians regarding the usefulness of ChatGPT in clinical practice and education (25). Our findings reinforce this generational pattern: students and residents reported significantly higher ChatGPT literacy and greater agreement in evaluating AI-generated content, whereas consultants approached such tools with greater caution. Notably, however, ethical awareness did not differ significantly across groups in our study, suggesting that discussions around privacy, risk, evidence verification, and professional responsibility have permeated all training levels. Although participants reported high levels of agreement with statements related to AI ethics, self-reported awareness does not necessarily reflect the ability to apply ethical principles in real clinical decision-making. Further educational initiatives should therefore emphasize applied ethical reasoning in the context of AI-assisted care.

The attitudes of surgical professionals in our study also parallel the results of Altowijri et al., who found moderate awareness and generally positive perceptions of AI among surgeons in Saudi Arabia, with younger and less experienced clinicians reporting the highest readiness to adopt AI. In both studies, familiarity with AI strongly aligned with perceived usefulness and willingness to integrate AI technologies into clinical practice (26). Our participants similarly reflected this trend: residents rated AI as more useful across nearly all surgical domains, while consultants remained more skeptical—particularly regarding intraoperative assistance. While this difference may partly reflect generational familiarity with digital technologies, alternative explanations should be considered. Less experienced participants may have limited exposure to the practical challenges, risks, and limitations of AI in real-world clinical settings, potentially leading to more optimistic perceptions. In contrast, consultants may draw on greater clinical experience, making them more attuned to issues such as reliability, accountability, workflow integration, and patient safety. Additionally, differences in medico-legal responsibility and decision-making burden may contribute to more cautious attitudes among senior clinicians (27, 28). These findings suggest that variation in attitudes toward AI is likely multifactorial, reflecting not only familiarity but also differences in clinical experience, responsibility, and risk perception.

Taken together, these findings highlight several key considerations for AI adoption in surgical settings. First, gaps in foundational AI perceived knowledge—particularly regarding system limitations, interpretability, and differences between algorithmic and traditional decision-making processes—suggest the need for structured and competency-based AI education across all training levels. Second, the strong interest in workshops and hands-on training reported by participants indicates that integrating AI literacy into existing medical curricula may improve confidence and reduce misconceptions. Third, concerns surrounding legal accountability, data privacy, and potential deskilling underscore the urgency of establishing institutional policies, clear regulatory frameworks, and ethical guidelines to support safe AI integration in surgical practice (29–32).

This study also offers meaningful regional insight, as empirical evidence on AI literacy and perceptions among surgical trainees and clinicians in the Middle East remains limited. By capturing differences across professional groups and identifying both readiness and hesitation, our findings contribute important data to guide the development of targeted training programs and organizational strategies for AI adoption in healthcare settings. Additionally, the measurement tools used in this study—including the Medical AI Literacy Scale (MAIL) and the ChatGPT Literacy Scale—reported strong internal reliability, providing a robust framework that can support future research on AI competencies among healthcare professionals.

Several limitations should be considered. The cross-sectional design captures perceptions at a single time point and may not reflect evolving attitudes as AI tools continue to develop and become more integrated into clinical practice. Given the cross-sectional design of the study, the observed relationships between AI exposure, AI literacy, and attitudes toward AI should be interpreted as associations rather than causal effects. Longitudinal or interventional studies would be required to determine whether increasing AI literacy directly influences attitudes or clinical use of AI technologies. Self-reported data may introduce social desirability or recall bias. Furthermore, although the sample was diverse across training levels, the findings are derived from a single national context, which may limit generalizability to other regions with differing levels of AI exposure or infrastructure. AI literacy levels may also be influenced by national factors such as digital infrastructure, regulatory frameworks, and institutional support for AI adoption in healthcare; as this study was conducted in Jordan, interpretation of the findings should consider the national context of AI implementation within healthcare systems. Given the unique nature of surgical workflows and the increasing emergence of AI applications in areas such as robotic surgery, imaging analysis, and intraoperative decision support, future studies may benefit from developing surgery-specific AI literacy instruments or domain-adapted subscales. AI literacy levels may also be influenced by national factors such as digital infrastructure, regulatory frameworks, and institutional support for AI adoption in healthcare; as this study was conducted in Jordan, interpretation of the findings should consider the national context of AI implementation within healthcare systems. Given the unique nature of surgical workflows and the increasing emergence of AI applications in areas such as robotic surgery, imaging analysis, and intraoperative decision support, future studies may benefit from developing surgery-specific AI literacy instruments or domain-adapted subscales. Future research should examine longitudinal changes in perceptions, the real-world impact of AI training programs, and how AI literacy influences actual clinical decision-making and patient outcomes.

## 5 Conclusion

This study highlights the current levels of AI and ChatGPT literacy, attitudes, and perceived utility among surgical consultants, residents, and medical students in Jordan. The findings suggest that residents and students report relatively higher engagement with AI tools and greater perceived usefulness across clinical domains, whereas consultants demonstrate more cautious attitudes, particularly regarding intraoperative applications. However, this relatively higher engagement among residents and students likely reflects educational or exploratory use of AI tools rather than independent real-world clinical application, given their level of training and supervision requirements.

Clinically, this indicates a need for structured AI and digital literacy training programs within surgical education to ensure safe and effective adoption of AI tools. Educational strategies may need to be tailored to different stages of training. For example, medical students may benefit from foundational AI concepts and critical appraisal skills, residents from practical integration of AI tools into clinical workflows, and consultants from advanced training focused on governance, ethical oversight, and clinical implementation.

Addressing barriers such as lack of knowledge, ethical concerns, and legal uncertainties can facilitate more confident integration of AI into clinical practice. The study also emphasizes the importance of including AI competencies as part of continuing medical education and surgical curricula to enhance decision-making, optimize patient outcomes, and mitigate risks associated with over-reliance on AI.

## Data Availability

The raw data supporting the conclusions of this article will be made available by the authors, without undue reservation.
