# Supplementary material for: Artificial intelligence and ChatGPT literacy among surgical healthcare professionals: knowledge, attitudes, and perceived clinical utility
Source: Front Digit Health. 2026 May 19;8:1766683. doi: 10.3389/fdgth.2026.1766683 (PMC13226627; doi:10.3389/fdgth.2026.1766683)
Supplement: Supplementary file 1 [file Datasheet1.docx]

**AI & ChatGPT in Surgery Literacy Questionnaire**

**Dear Participant,**

You are invited to take part in a research study exploring artificial intelligence (AI) literacy and the use of AI tools such as ChatGPT among Surgical medical students, Surgery residents, and Surgery specialists. The purpose of this study is to assess current knowledge, attitudes, experiences, and perceived barriers related to the integration of AI in surgical education and clinical practice. The survey includes both validated and newly developed items and will take approximately 15–20 minutes to complete.

Your participation is entirely voluntary, and all responses will remain confidential and anonymized. You may choose to skip any question or withdraw at any point without any consequences. By proceeding with this questionnaire, you are providing your informed consent to participate in this study.

This document was reviewed and approved by the Institutional Review Board (IRB) at King Hussein Cancer Center. If you have any general questions or questions related to your rights as a participant in research, please call the IRB office at 5300460 Ext. 1669 or Dr. Yacoub Yousef at 0777647272. If you have any questions about the study, please feel free to contact the principal investigator Yasmin Safi, Email: YS.11060@KHCC.JO

Thank you for your valuable contribution to this research.

**SECTION A: Demographics & Professional Background**

1. Age: _______
2. Gender: ☐ Male ☐ Female ☐ Prefer not to say
3. Current Position:
   ☐ Medical Student
   ☐ Resident
   ☐ Specialist
   ☐ Other: __________
4. Specialty (if applicable): _______________
5. Years of Clinical/Academic Experience: _______________
6. Have you received any formal training in Artificial Intelligence?
   ☐ Yes ☐ No
7. How would you rate your understanding of the following AI concepts?

| **Concept** | **Never Heard = 1** | **Heard of It = 2** | **Understand Basics = 3** | **Understand Well = 4** | **Can Apply = 5** |
| --- | --- | --- | --- | --- | --- |
| Machine Learning (ML) | ☐ | ☐ | ☐ | ☐ | ☐ |
| Deep Learning (DL) | ☐ | ☐ | ☐ | ☐ | ☐ |
| Computer Vision (CV) | ☐ | ☐ | ☐ | ☐ | ☐ |
| Robotic Surgery AI | ☐ | ☐ | ☐ | ☐ | ☐ |
| Clinical Decision Support AI | ☐ | ☐ | ☐ | ☐ | ☐ |
| Natural Language Processing (NLP) | ☐ | ☐ | ☐ | ☐ | ☐ |

1. Have you used ChatGPT (or similar AI tools) for medical or academic purposes?
   ☐ Yes ☐ No
   If yes, please describe briefly: _________________________

**SECTION B: Meta-AI Literacy Scale (Quantitative - 5-point Likert scale)**
*Please indicate your level of agreement with the following statements:*
(1 = Strongly Disagree; 5 = Strongly Agree)

**B.1: Use & Apply**

1. I know how to use AI tools in surgical or medical settings.
2. I have used AI applications to support clinical decision-making.
3. I feel confident in integrating AI tools in my clinical practice.
4. I can identify appropriate clinical scenarios where AI could be useful.
5. I am able to critically assess AI-based recommendations.
6. I have actively engaged with AI tools for learning or practice.

**B.2: Know & Understand**
7. I understand how AI works at a basic level.
8. I am familiar with different types of AI used in healthcare.
9. I understand the limitations of AI systems.
10. I can explain how machine learning differs from traditional programming.
11. I can distinguish between AI, automation, and robotics in medicine.
12. I understand how data quality affects AI performance.

**B.3: Detect AI**
13. I can recognize when AI is being used in clinical tools.
14. I can differentiate between AI-generated and human-generated content.
15. I know when a decision is based on AI algorithms.

**B.4: AI Ethics**
16. I am aware of ethical concerns related to using AI in surgery.
17. I understand the importance of data privacy and confidentiality in AI applications.
18. I believe ethical guidelines should be mandatory in AI tool implementation.

**SECTION C: ChatGPT Literacy (Quantitative - 5-point Likert scale)**
*Please indicate your level of agreement with the following statements:*
(1 = Strongly Disagree; 5 = Strongly Agree)

1. I frequently use ChatGPT (or similar tools) in my studies/work.
2. I understand the types of tasks ChatGPT is good at.
3. I am aware of the limitations and potential errors of ChatGPT.
4. I believe ChatGPT is useful for clinical or educational purposes.
5. I am concerned about the reliability of ChatGPT outputs in a clinical context.
6. I believe ChatGPT can enhance surgical education.
7. I feel confident evaluating the quality of ChatGPT’s responses.
8. I understand the ethical considerations of using ChatGPT in clinical settings.

**SECTION D: Attitudes and Barriers Toward AI Integration (Quantitative)**
*Please indicate your level of agreement with the following statements:*
(1 = Strongly Disagree; 5 = Strongly Agree)

1. I am willing to integrate AI into my clinical practice.
2. My institution supports AI training or use in clinical settings.
3. I feel that I need more training to confidently use AI tools.
4. I am concerned about legal or regulatory risks when using AI.
5. I believe AI can improve surgical outcomes.
6. I worry that AI could lead to over-reliance or deskilling of physicians.
7. I think AI literacy should be a core part of surgical education
8. I am interested in attending AI-related training sessions or workshops

How do you perceive AI in the following areas?

| **Domain** | **Very Harmful** | **Harmful** | **Neutral** | **Beneficial** | **Very Beneficial** |
| --- | --- | --- | --- | --- | --- |
| Surgical planning | ☐ | ☐ | ☐ | ☐ | ☐ |
| Diagnostic accuracy | ☐ | ☐ | ☐ | ☐ | ☐ |
| Intraoperative decision-making | ☐ | ☐ | ☐ | ☐ | ☐ |
| Surgical robotics | ☐ | ☐ | ☐ | ☐ | ☐ |
| Medical documentation | ☐ | ☐ | ☐ | ☐ | ☐ |

**SECTION E: Barriers and Concerns (Quantitative)**

1. What do you believe are the **main barriers** to AI adoption in surgery in your region? (Select up to 3)
   ☐ Lack of knowledge/training
   ☐ Ethical concerns
   ☐ Legal/liability issues
   ☐ Cost/financial burden
   ☐ Lack of infrastructure
   ☐ Data privacy risks
   ☐ Resistance from senior staff
2. What ethical concerns worry you most?
   ☐ Algorithmic bias
   ☐ Data misuse/privacy
   ☐ Replacing human judgment
   ☐ Lack of accountability
   ☐ Other: __________
